# Intensive Care Medicine Science: An Art Based on Applied Physiology?

**DOI:** 10.1155/2015/479134

**Published:** 2015-03-10

**Authors:** Karim Bendjelid, Bruno Levy, Alain Broccard

**Affiliations:** ^1^Geneva Medical School, 1211 Geneva, Switzerland; ^2^Intensive Care Service, Geneva University Hospitals, Geneva Medical School, 1211 Geneva, Switzerland; ^3^Service de Réanimation Médicale Brabois, Vandoeuvre lès Nancy, France; ^4^University of Minnesota, Regions Hospital Pulmonary and Critical Care Division, 640 Jackson Street, St. Paul, MN 55455, USA

The provision of complex medical care in the intensive care unit (ICU) environment is challenging as the established goals of intensive care are to reduce the morbidity and mortality related to critical illness, maintain organ function, and restore health. Despite technological advances, the prevalence of death in the ICU remains a serious problem. Even if it is unlikely that intensive care medicine is recognized as an evidence-based process, the present reality reveals that scientific proof regarding patient management is very rare [1]. However, this medicine is pathophysiology based, and it necessitates the monitoring of physiological abnormalities to treat the patient. Indeed, outcome prediction models measuring the severity of illness of patients admitted to the ICU use physiological variables to predict hospital mortality. For instance, from the clinical and medical management perspective, the state-of-the-art Simplified Acute Physiology Score (SAPS) model is frequently used.

In the present state of medical knowledge, it is important to account for human physiology when treating critically ill patients, as these patients often present with circulatory failure. Understanding the properties of cardiovascular physiology is an excellent example of how general principles are useful in comprehending hemodynamic instability and shock states [2]. Therefore, from a physiological point of view, mastering the cardiovascular physiology and identifying the pathophysiological mechanisms and archetype involved in shock allow the physician to manage each situation according to the specific characteristics of the state of the patient.

In the first half of the 20th century, there was considerable confusion among physiologists regarding the combined role of the heart and vasculature in determining flow and pressure in the cardiovascular system [3]. Currently, our understanding of these subjects is constantly evolving. As part of the present special issue, respected researchers and physiologists provide an outline of the important advances in cardiovascular physiology and the great progress made in recent years within this discipline. The present papers take a deeper look at selected physiological principles and main beliefs that are the basis of intensive care medicine. In fact, the present papers investigate physiological principles using various techniques described in humans and animal experimentation. In addition, in the present issue, the use of mathematical and simulated models as alternative methods is highlighted as a potential way to study specific cases in diverse vessels. These papers, which describe numerical simulations of flow, ranging from the geometry of steady flow in rigid vessels to unsteady flow with elastic vessel walls, deserve further attention as several flow and wall models are compared. Finally, all of these works reinforce our use of these principles in clinical decision-making.

Thus, it must be established and accepted that specific cardiovascular physiology concepts are essential if and only if an appropriate form of intervention or treatment could be administered based on this knowledge. Indeed, it is important to note that our way of thinking about cardiovascular physiology in the ICU may sometimes be imperfect as views and understanding need to evolve [4]. Certainly, in the present state of knowledge, the most significant advantage of mastering cardiovascular physiology in critically ill patients is the capability to recommend new methods of treatment for shock.

*Karim Bendjelid* *Bruno Levy* *Alain Broccard*
